# Quantitative Distribution of DNA, RNA, Histone and Proteins Other than Histone in Mammalian Cells, Nuclei and a Chromosome at High Resolution Observed by Scanning Transmission Soft X-Ray Microscopy (STXM)

**DOI:** 10.3390/cells8020164

**Published:** 2019-02-16

**Authors:** Kunio Shinohara, Shigenobu Toné, Takeo Ejima, Takuji Ohigashi, Atsushi Ito

**Affiliations:** 1 School of Engineering, Tokai University, Hiratsuka, Kanagawa 259-1292, Japan; kshino-tky@umin.net; 2 School of Science and Engineering, Tokyo Denki University, Hatoyama, Saitama 350-0394, Japan; tone@mail.dendai.ac.jp; 3 Institute of Multidisciplinary Research for Advanced Materials, Tohoku University, Sendai 980-8577, Japan; takeo.ejima.e7@tohoku.ac.jp; 4 UVSOR Synchrotron, Institute Molecular Science, Okazaki, Aichi 444-8585, Japan; ohigashi@ims.ac.jp

**Keywords:** X-ray spectromicroscopy, STXM, molecular mapping, quantitative study, local content, mammalian cell, apoptotic cell, chromosome

## Abstract

Soft X-ray microscopy was applied to study the quantitative distribution of DNA, RNA, histone, and proteins other than histone (represented by BSA) in mammalian cells, apoptotic nuclei, and a chromosome at spatial resolutions of 100 to 400 nm. The relative distribution of closely related molecules, such as DNA and RNA, was discriminated by the singular value decomposition (SVD) method using aXis2000 software. Quantities of nucleic acids and proteins were evaluated using characteristic absorption properties due to the 1s–π * transition of N=C in nucleic acids and amide in proteins, respectively, in the absorption spectra at the nitrogen K absorption edge. The results showed that DNA and histone were located in the nucleus. By contrast, RNA was clearly discriminated and found mainly in the cytoplasm. Interestingly, in a chromosome image, DNA and histone were found in the center, surrounded by RNA and proteins other than histone. The amount of DNA in the chromosome was estimated to be 0.73 pg, and the content of RNA, histone, and proteins other than histone, relative to DNA, was 0.48, 0.28, and 4.04, respectively. The method we present in this study could be a powerful approach for the quantitative molecular mapping of biological samples at high resolution.

## 1. Introduction

In principle, X-ray microscopy has the following advantages for the observation of biological samples over other microscopic methods: higher resolution than optical microscopy with respect to the diffraction limit; good absorption contrast in hydrated conditions with soft X-rays in an energy range, the so-called water window; better transmittance than electron microscopy; and the discrimination of biological molecules by spectromicroscopy, the combination of microscopy and spectroscopy using absorption of fine structures in biomolecules according to the energies of carbon, nitrogen, and oxygen absorption edges. These advantages have been demonstrated by the development of X-ray microscopes [1] with absorption contrast [2,3], followed by phase contrast with coherent diffraction imaging and ptychographic imaging [4,5,6], and include direct observation of intact biological samples under hydrated conditions at a resolution of less than 100 nm with contact microscopy [7,8]; mapping elements in biological samples [9,10]; 3D observation of frozen hydrated specimens [11,12,13,14,15,16,17]; and the mapping of molecular distributions by spectromicroscopy [18,19,20,21,22,23,24]. Spectromicroscopy is a powerful tool for the analysis of local quantities and distribution of biomolecules.

The characteristic nature of the X-ray absorption spectrum near the absorption edge of each element—called near-edge X-ray absorption fine structure (NEXAFS)—is useful for the separation of constituent molecules in biological specimens because NEXAFS depends on the chemical structures of the molecules. NEXAFS at the nitrogen K absorption edge shows clear separation of the absorption spectra of nucleic acids and proteins, and it has been applied to identify these molecules in images of a mammalian cell and a chromosome observed with a scanning transmission soft X-ray microscope (STXM) [23,24], a microscopy technique in which samples are moving on the focused soft X-ray beam. In addition, combined NEXAFS at the K absorption edges of carbon, nitrogen, and oxygen was found to be useful for differentiating closely related molecules, such as DNA and RNA [24]. In the present study, we applied NEXAFS at the carbon, nitrogen, and oxygen K absorption edges to analyze molecular distributions in mammalian cells, apoptotic nuclei, and a chromosome. We found interesting quantitative distributions of biomolecules in these samples, with clear localization of RNA. Thus, the method described here should provide a powerful tool to resolve molecular distributions in biological systems without any labeling assistance at high resolution.

## 2. Materials and Methods

CHO cells at interphase or mitotic (M) phase were prepared as described previously [23,24]. HeLa S3 cells were grown in RPMI 1640 medium supplemented with 10% fetal bovine serum and antibiotics at 37 °C in a humidified 5% CO_2_ atmosphere. For the study of apoptotic nuclei, HeLa S3 nuclei were prepared as described previously [25]. 

For the observation of CHO and HeLa S3 cells, these cells were plated and grown on a silicon nitride (Si_3_N_4_) membrane (thickness, 100 nm; purchased from Silson Ltd., Warwickshire, England) and processed using a previously described method [24]. Mitotic CHO cells were treated as previously described [23]. Intact and apoptotic nuclei were fixed with 2.5% glutaraldehyde, attached to a formvar membrane covered with Corning^®^ Cell-Tak Cell Tissue Adhesive, washed with an increasing concentration of ethanol, and dried. Molecules including calf thymus histone (Type II-A, Product No. H9250) were obtained from Sigma-Aldrich Co. (St. Louis, MO, USA), and they were prepared according to previously described methods [24].

**Figure 1:**
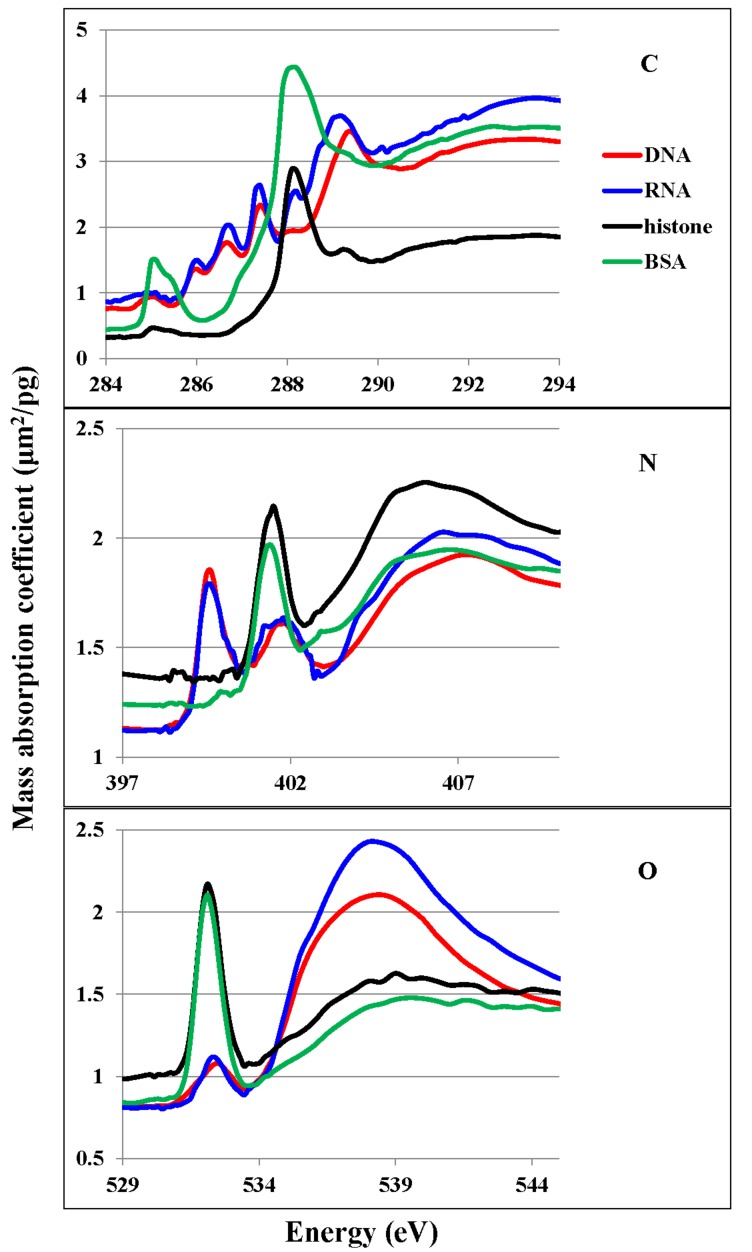
NEXAFS spectra of DNA (red line), RNA (blue line), histone (black line), and BSA (green line) at the carbon (C), nitrogen (N), and oxygen (O) K absorption edge regions.

Observation and data collection for the cells and molecules were performed as previously described [23] at a focus size of 75 nm in diameter, with vertical and horizontal steps of 400 and 400 nm for a CHO cell at interphase or M phase, 100 and 100 nm for a chromosome, 200 and 200 nm for a HeLa S3 cell, and 120 and 120 nm for an intact or apoptotic nucleus, respectively. Step sizes were determined based on limitations of the stability of the imaging system for the observation sizes of the regions of interest. These step sizes were larger than the focus size of the beam and defined the resolution of the image. We followed the method previously described [23] for the quantitative analysis of nucleic acids and proteins using NEXAFS spectra at the nitrogen K absorption edge (see middle panel of Figure 1. The peak is assigned to the absorption of N=C in nucleic acids at 399.6 eV and amide in proteins at 401.4–401.5 eV [26]).

The results were analyzed using the program aXis2000 [27]. We assumed that the measured spectra of DNA, RNA, histone, and BSA are representative of the spectra of DNA, RNA, histone, and proteins other than histone [18,19], respectively. Calculations were made according to previously described methods [23,24]. Briefly, each energy stack file of cell images and spectrum file of DNA, RNA, histone, or BSA at the carbon, nitrogen, and oxygen K absorption edge regions was combined to form one stack file and one absorption spectrum, respectively. The combined image stack file was named spect1. DNA and RNA images were extracted from spect1 using the SVD method of the aXis2000 program. The image for the ratio of DNA/(DNA + RNA) was applied to an image that represented the difference between the images obtained at 399.6 and 398 eV (the peak height in the NEXAFS of nucleic acids, i.e., DNA and RNA) in spect1 for the separation of the DNA image. The DNA spectrum was normalized by the peak height at 399.6 eV and used to generate an energy stack file (named DNAspect) with the DNA image. DNAspect was subtracted from spect1, resulting in a stack file named spect2D. Subsequently, an RNA image was obtained from the peak height at 399.6 eV in the spect2D. The RNA spectrum was normalized by the peak height at 399.6 eV and used to generate an energy stack file (named RNAspect) with the RNA image. RNAspect was subtracted from spect2D, resulting in a stack file named spect2R. Similarly, histone and BSA images were extracted from spect2R using the SVD method of aXis2000. The image for the ratio of histone/(histone + BSA) was applied to an image that represented the difference between the images at 401.5 and 398 eV (the peak height in the NEXAFS of histone) in spect2R for the separation of the histone image. The histone spectrum was normalized by the peak height at 401.5 eV and used to generate an energy stack file (named histone-spect) with the histone image. Histone-spect was subtracted from spect2R, resulting in a stack file named spect2H. Then, the BSA image was obtained from the peak height at 401.4 eV (the difference between the images at 401.4 and 398 eV) in spect2H. The BSA spectrum was normalized by the peak height at 401.4 eV, and used to generate an energy stack file (named BSAspect) with the BSA image. BSAspect was subtracted from spect2H, resulting in a stack file named spect3. 

In quantitative estimates, optical density (OD) images at 398 eV in DNAspect, RNAspect, histone-spect, or BSAspect were converted to mass thickness, defined as mass per unit area, by using the mass absorption coefficients of DNA, RNA, histone, and BSA at 398 eV, calculated from the database for mass absorption coefficients of constituent elements [28]. For comparison of the structure of each local spectrum, spectra were normalized as described previously [23].

## 3. Results

### 3.1. NEXAFS of DNA, RNA, Histone, and BSA

Figure 1 shows the NEXAFS profiles for the mass absorption coefficients of DNA, RNA, histone, and BSA at the carbon, nitrogen, and oxygen K absorption edge regions. The assignments of absorption peaks for DNA and BSA have been reported elsewhere [26]. For the analysis, it is important to note that there is an independent absorption peak of 1s to π * transition of N=C in the base of nucleic acids at 399.6 eV, which is clearly separated from a peak of 1s to π * transition of amide in proteins at around 401.4 eV. Since nucleic acids and proteins are the main nitrogen-containing components in biological cells, these peaks are important markers for the quantitative analysis of these molecules.

### 3.2. Absorption Images

**Figure 2:**
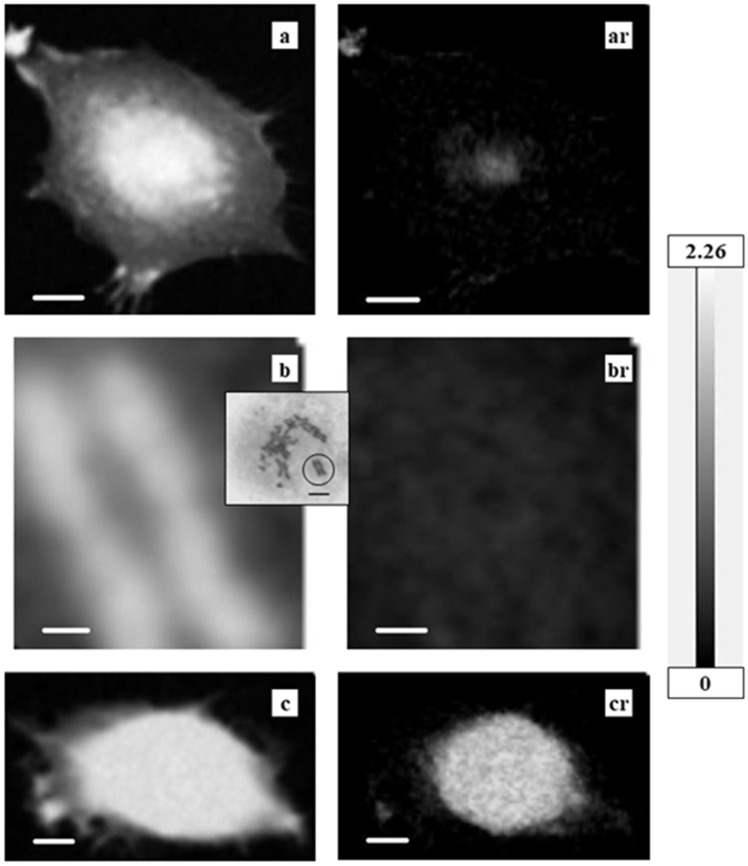
Absorption images on the left (spect1), with residual images after subtraction of nucleic acids and proteins (spect3) on the right. (**a**, **ar**) CHO cell; (**b**, **br**) chromosome—location indicated by circle in (**b inset**), which shows a CHO cell at M phase; (**c**, **cr**) HeLa S3 cell. All observed at 290 eV. Grayscale on the right indicates OD, which is consistent among these images to allow for comparison of observed and residual images. Scale bars represent 5 μm (**a**, **ar**), 0.5 μm (**b**, **br**), and 2 μm (**c**, **cr**).

**Figure 3:**
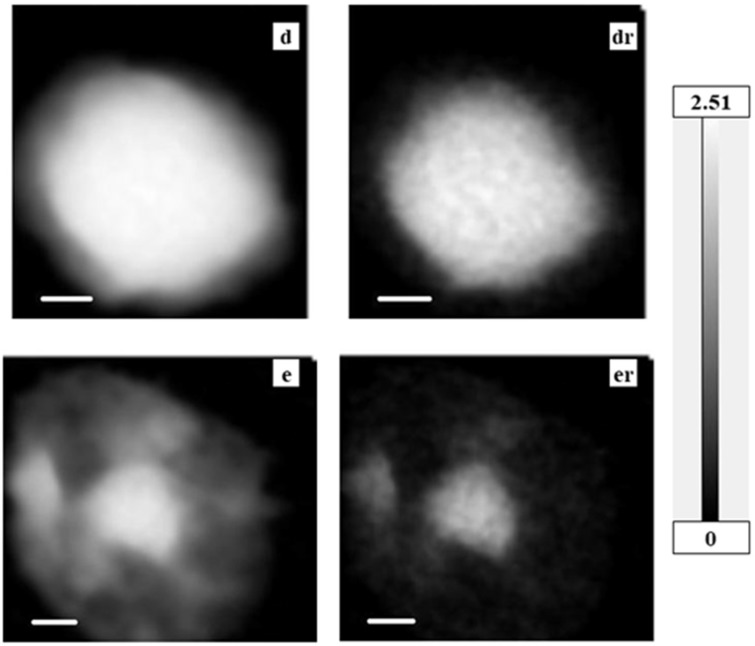
Absorption images on the left (spect1), with residual images after subtraction of nucleic acids and proteins (spect3) on the right. (**d**, **dr**) an isolated nucleus; (**e**, **er**) an apoptotic nucleus. All observed at 398 eV. Grayscale on the right indicates OD, which is consistent among these images to allow for comparison of observed and residual images. Scale bars represent 1 μm.

**Figure 4:**
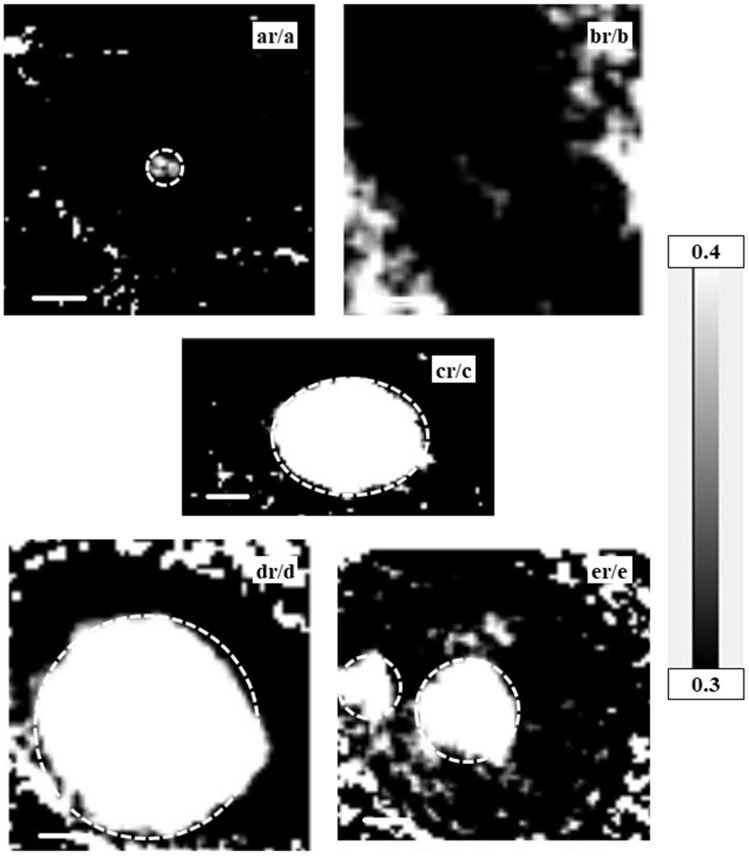
Ratio images of each residual-to-observed image pair shown in Figure 2 and Figure 3. Each residual image (spect3) was divided by the absorbed image (spect1) for (**ar**/**a**) the CHO cell; (**br**/**b**) the chromosome; (**cr**/**c**) the HeLa S3 cell; (**dr**/**d**) the isolated nucleus; and (**er**/**e**) the apoptotic nucleus. Grayscale on the right indicates the residual-to-absorbed image OD ratio of residual image to absorbed image for values higher than 0.3. Scale bars are 5 μm (**ar**/**a**), 0.5 μm (**br**/**b**), 2 μm (**cr**/**c**), and 1 μm (**dr**/**d** and **er**/**e**). Areas possibly unreliable for analysis are indicated with dashed circles.

Observed absorption images (spect1) and residual images after subtraction of nucleic acids and proteins (spect3) are shown in Figure 2, Figure 3 and Figure 4 for a CHO cell at interphase (Figure 2a, ar); a CHO cell at M phase (Figure 2b inset) with an enlarged image of a chromosome (Figure 2b, br indicated by a circle in the inset image); a HeLa S3 cell (Figure 2c, cr); and nuclei from HeLa S3 cells in both intact (Figure 3d, dr) and apoptotic stages (Figure 3e, er). Energies of these images were selected for clear contrast. Grayscales are consistent across images to allow for comparison of the observed images with the residual images. Figure 4 shows the ratio images of each residual image (spect3) to each observed image (spect1). Since the relative amount of nucleic acids and proteins as a proportion of total biological molecules in a cell is estimated to be around 73% by weight [29], the residual image in the ratio image should be less than 30%. Therefore, the analysis of images with a ratio higher than 0.3 may not be reliable because the presence of nucleic acids and/or proteins may not be subtracted in full from the original absorption, probably due to insufficient flux for the observation. However, this does not hold for the outside of a chromosome (position 4 in Figure 11) where the content of nucleic acids and proteins is expected to be low. Those areas that may potentially be unreliable for analysis are indicated, wherever they occur, with a dashed circle.

### 3.3. Quantitative Distribution of DNA, RNA, Histone, and Proteins Other Than Histone

**Figure 5:**
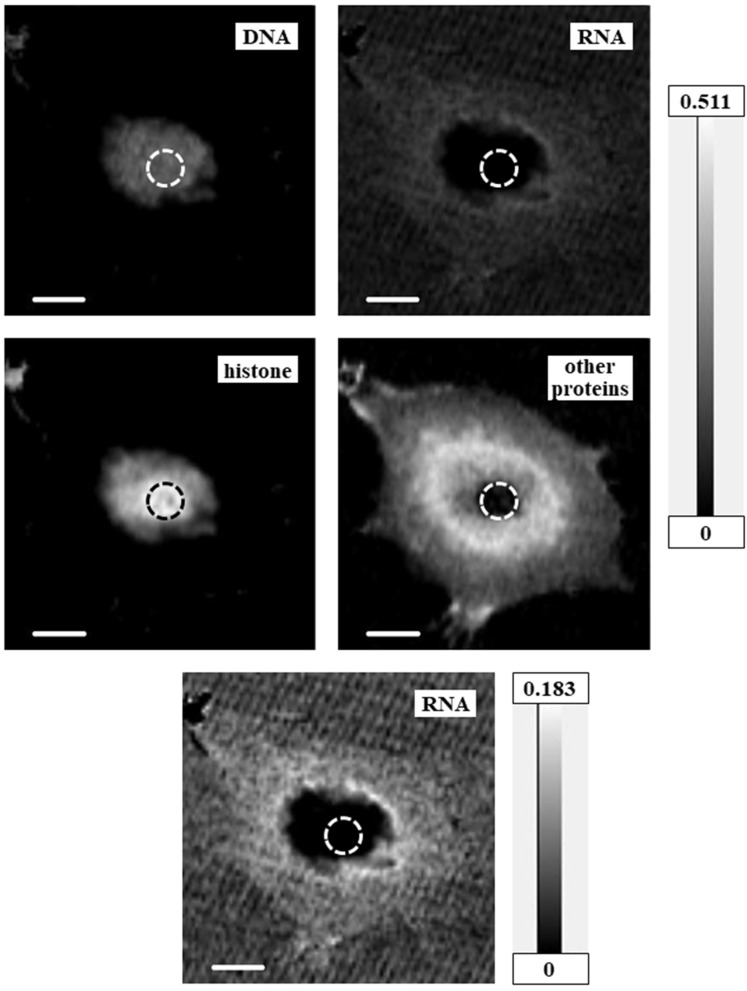
Mass thickness images for DNA, RNA, histone, and proteins other than histone of the CHO cell. The top four images use the same grayscale (in units of pg/μm^2^) for comparison of each level. Bottom image shows the distribution of RNA with a different grayscale (pg/μm^2^) for clarity. Scale bars = 5 μm. Areas possibly unreliable for analysis are encircled by dashed lines.

**Figure 6:**
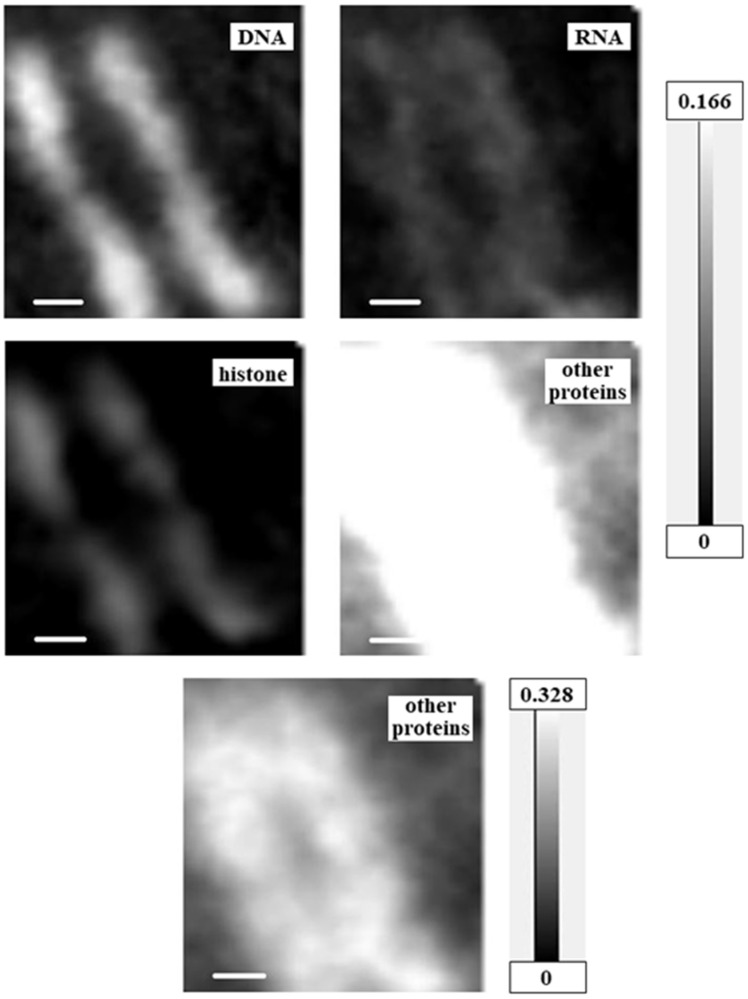
Mass thickness images for DNA, RNA, histone, and proteins other than histone of the chromosome. Grayscales on the right represent units of pg/μm^2^—scale is consistent for the top four images, and different for the bottom image to show the distribution of other proteins more clearly. Scale bars represent 0.5 μm.

**Figure 7:**
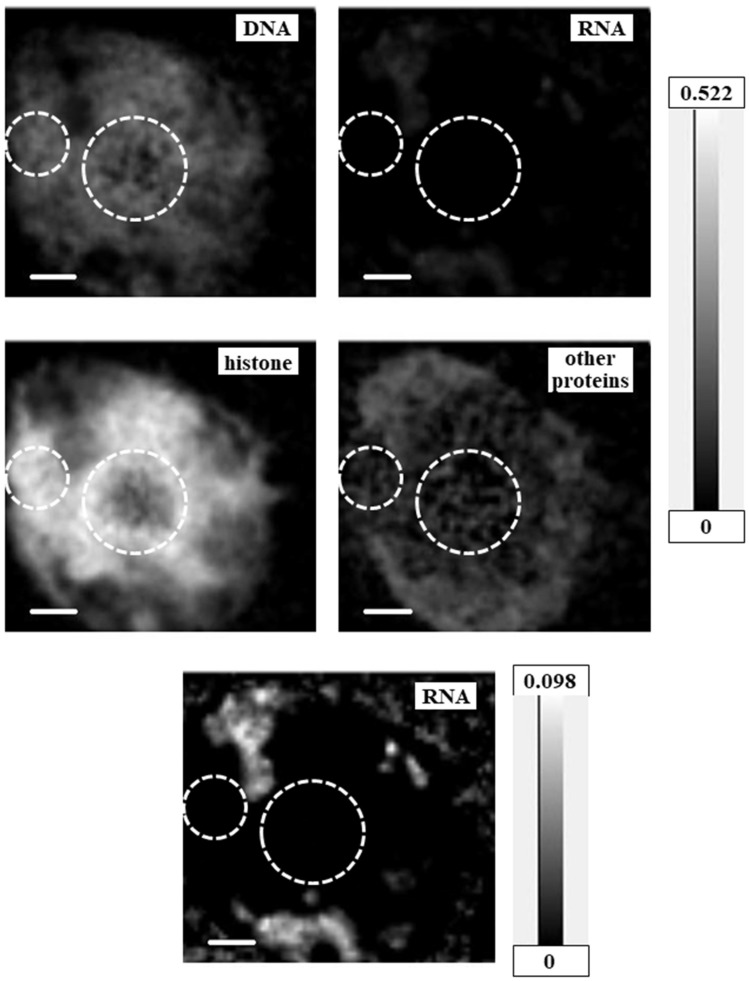
Mass thickness images for DNA, RNA, histone, and proteins other than histone of the apoptotic nucleus. Grayscales on the right represent units of pg/μm^2^—scale is consistent for the top four images, and different for the bottom image to show the distribution of RNA more clearly. Scale bars represent 1 μm. Areas possibly unreliable for the analysis are encircled with dashed lines.

Two-dimensional quantitative distributions of molecules were calculated as mass thickness, i.e., total mass at the unit area (pg/μm^2^), by the method described in Materials and Methods using aXis2000 software (Figure 5, Figure 6 and Figure 7 and Supplementary Figures S1–S3). Levels of proteins other than histone were estimated by the absorption level of BSA at the amide peak in the NEXAFS of the nitrogen K absorption edge region. Since the amide peak corresponds to the absorption of proteins, the estimated levels should be correct, even though BSA is not a component of a cell and the spectral feature may be modified. In addition, for the RNA images in Figure 5 and Figure 7, Supplementary Figures S2 and S3, and the images of proteins other than histone in Figure 6, we used different grayscales to the distribution for clarity. Supplementary Figure S1 shows combined images of DNA and RNA; DNA and histone; and DNA, RNA, and total proteins (i.e., histone plus proteins other than histone) in comparison with the image of DNA.

### 3.4. Color Expression of the Molecular Distribution

**Figure 8:**
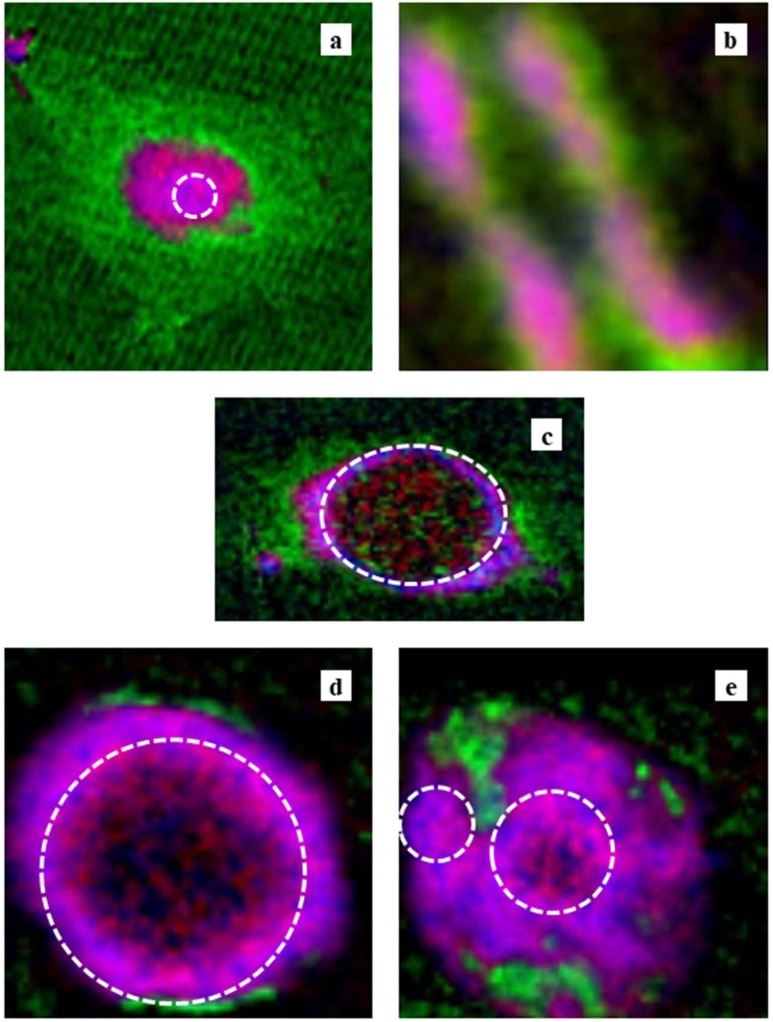
RGB expression of the images of the (**a**) CHO cell, (**b**) chromosome, (**c**) HeLa S3 cell, (**d**) isolated nucleus, and (**e**) apoptotic nucleus. DNA, RNA, and histone are displayed as red, green, and blue, respectively. Areas possibly unreliable for the analysis are encircled by dashed lines.

**Figure 9:**
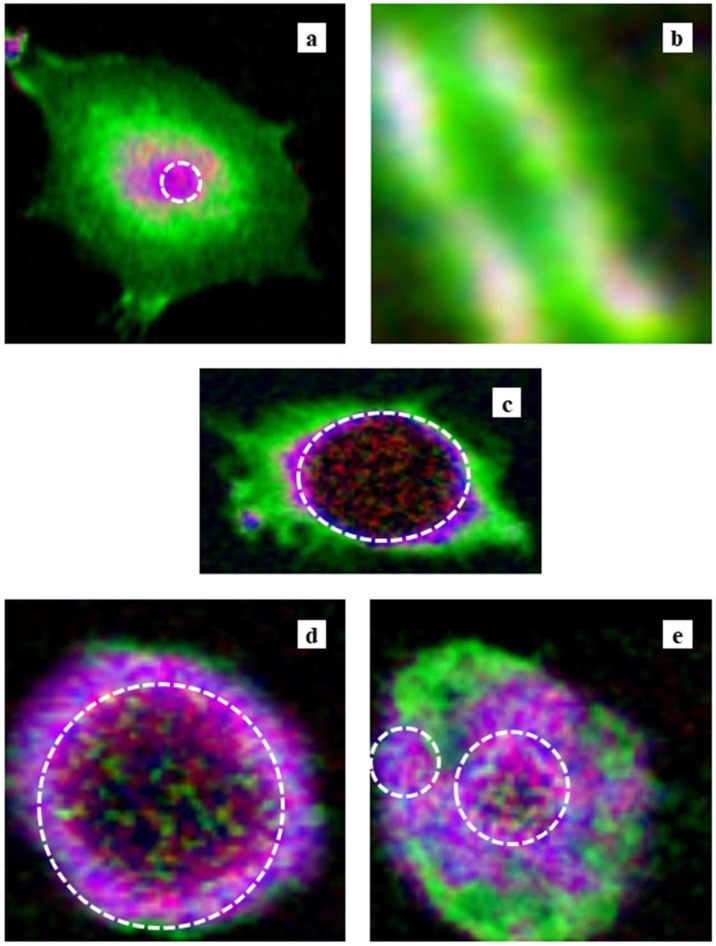
RGB expression of the images of the (**a**) CHO cell, (**b**) chromosome, (**c**) HeLa S3 cell, (**d**) isolated nucleus, and (**e**) apoptotic nucleus. DNA, proteins other than histone, and histone are displayed as red, green, and blue, respectively. Areas possibly unreliable for the analysis are encircled by dashed lines.

Based on mass thickness, images of RGB expression for the distribution of DNA (red), RNA (green), and histone (blue) in the CHO cell are shown in Figure 8, whereas Figure 9 shows DNA (red), proteins other than histone (green), and histone (blue). We observed that DNA and RNA in cells were distributed mainly in the nucleus and cytoplasm, as shown in Figure 8a (CHO) and 8c (HeLa S3). RNA was not clearly observed in the nucleus of the CHO cell (Figure 8a), the nucleus of the HeLa S3 cell (Figure 8c), or the intact nucleus (Figure 8d), except in the apoptotic nucleus (Figure 8e). In the apoptotic nucleus, RNA was clearly localized in a different area than DNA or histone (Figure 8e). In the area where RNA was observed, proteins other than histone were also mainly distributed (Figure 9e). Histone was found in the nucleus, either with or without DNA, in all the images. Proteins other than histone were distributed in an area similar to RNA, as well as in a wide area including the nucleus (Figure 9). In the chromosome, DNA and histone were found in a core position, surrounded by RNA and proteins other than histone (Figure 8b and Figure 9b).

### 3.5. Local Spectra of Absorption

**Figure 10:**
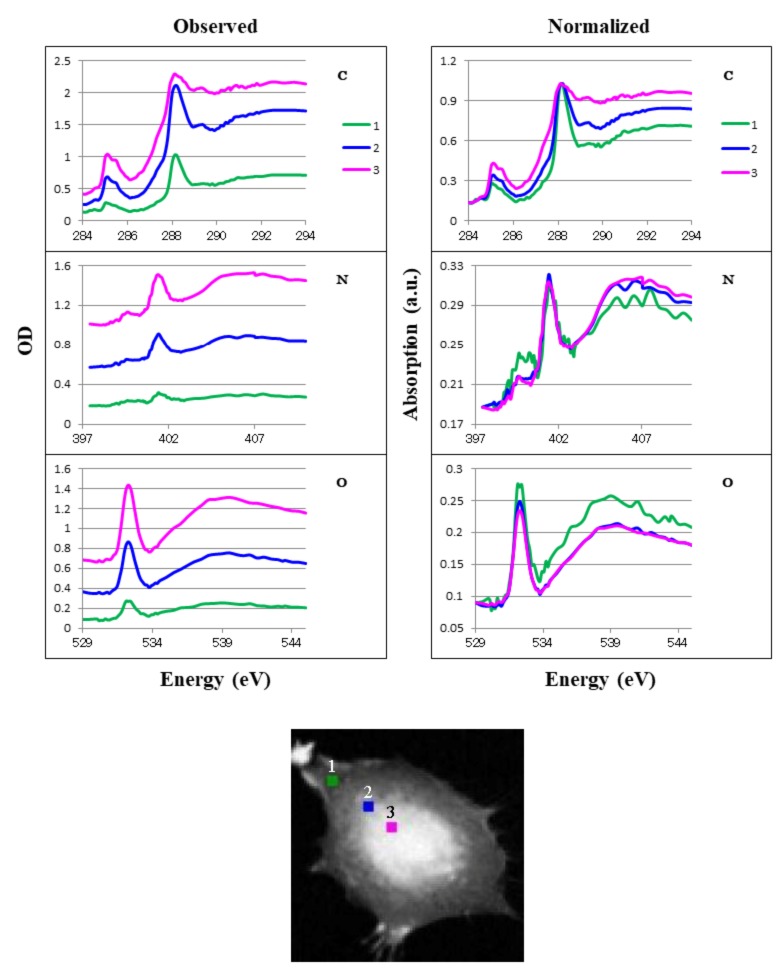
Absorption spectra of local areas in the CHO cell. Positions 1–3 are shown in the lower figure (area: 4 × 4 pixels each). Observed data are shown on the left, normalized data on the right. Numbers correspond to the positions of cytoplasm (1), cytoplasm near the nucleus (2), and the nucleus near its edge (3). C, N, and O are photon energy regions of the K absorption edges for carbon, nitrogen, and oxygen, respectively.

**Figure 11:**
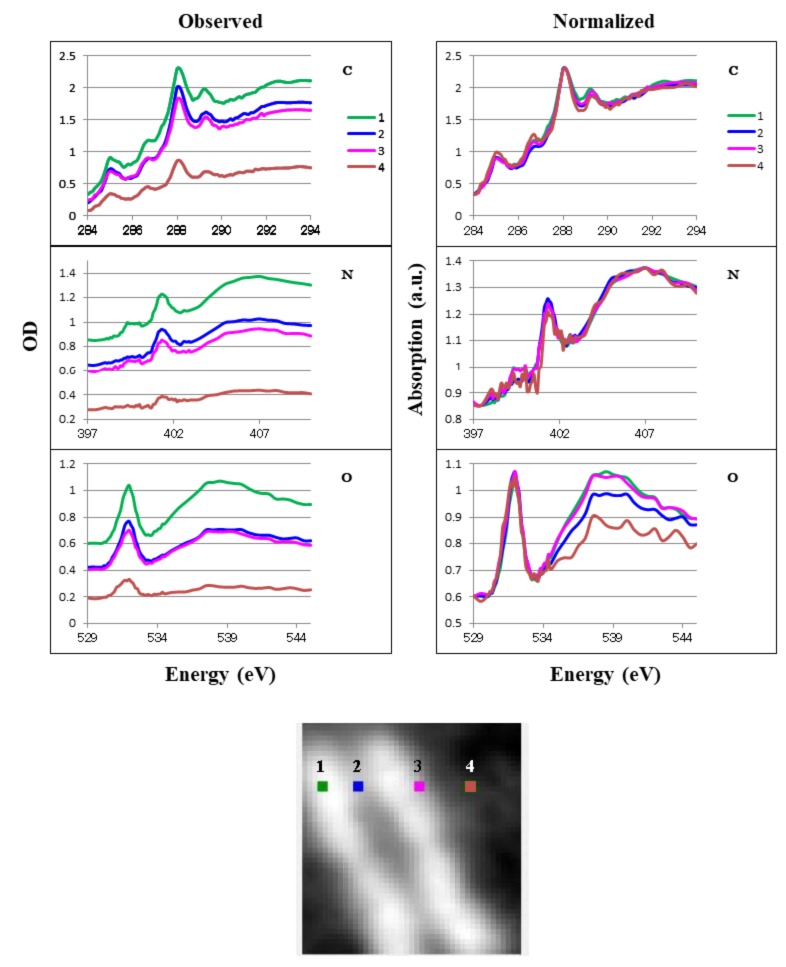
Absorption spectra of local areas in the chromosome. Positions 1–4 are shown in the lower figure (area: 2 × 2 pixels each). Observed data are shown on the left, normalized data on the right. Numbers correspond to the positions in the left arm (1), between the arms (2), in the right arm (3), and outside the chromosome (4). C, N, and O are photon energy regions of the K absorption edges for carbon, nitrogen, and oxygen, respectively.

**Figure 12:**
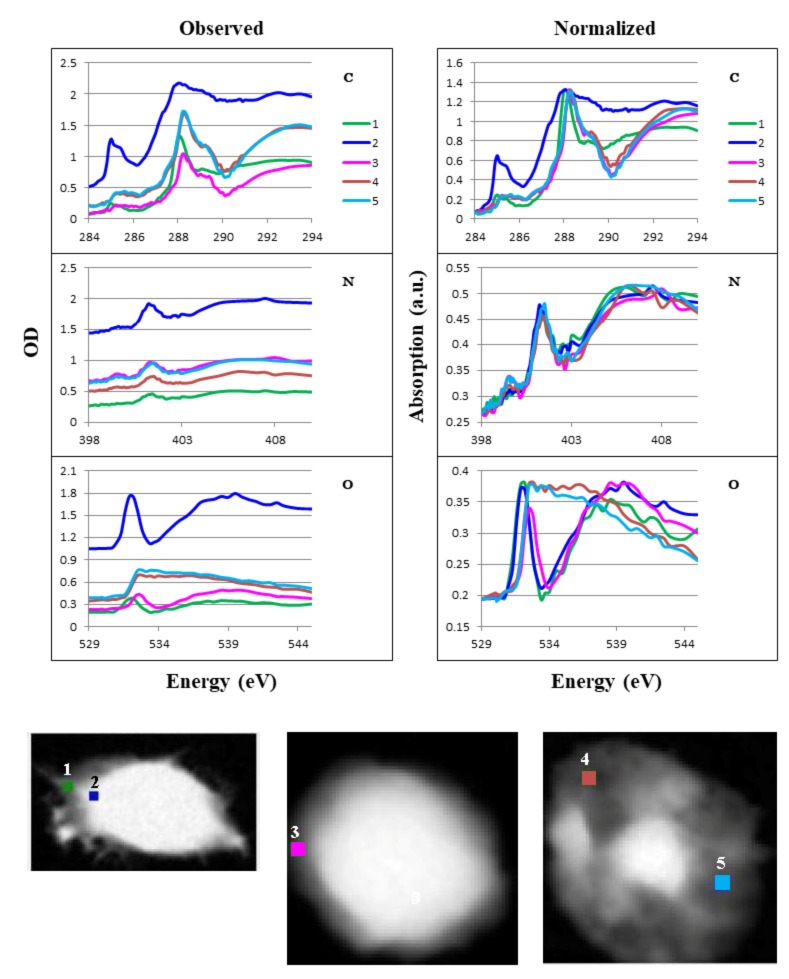
Absorption spectra of local areas in the HeLa S3 cell, isolated nucleus, and apoptotic nucleus. Positions 1–5 are shown in the lower figure (area: 4 × 4 pixels each). Observed data are shown in left panel, normalized data on the right. Numbers correspond to the positions of cytoplasm (1) and nucleus near its edge (2) in the HeLa S3 cell; nucleus near its edge in the isolated nucleus (3); and two different positions in the apoptotic nucleus (4, 5). C, N, and O are photon energy regions of the K absorption edges for carbon, nitrogen, and oxygen, respectively.

We obtained local absorption of spect1 at three positions in the CHO cell (Figure 10), four positions in the chromosome (Figure 11), and five positions in the HeLa S3 cell, including one in the overall cell, two in the isolated intact nucleus, and two in the apoptotic nucleus (Figure 12). Spectra for the positions in areas where the absorption ratio was higher than 0.3 in the ratio images (Figure 4) were excluded. We compared observed absorption levels among the local areas and normalized the results for further comparison (Figure 10, Figure 11 and Figure 12 L/R panels). A similar structure was found among all positions except positions 4 and 5 in the apoptotic nucleus (Figure 12, where highly different spectra were found, especially in the oxygen K absorption edge region. It should be noted that the spectra in the chromosome showed little difference to the profiles at all the positions 1 to 4 (Figure 11 Normalized), although the molecular contents are significantly different among those positions (Table 1). This result suggests that spectral differences in observed images may be less sensitive to the distribution of biomolecules.

### 3.6. Estimation of Local Quantities

Quantities of DNA, RNA, histone, and other proteins were obtained from Figure 5, Figure 6 and Figure 7 and Supplementary Figures S2 and S3 for the local areas shown in Figure 10, Figure 11 and Figure 12 and the whole image area of the chromosome, and are summarized in Table 1. These results show the clear quantitative differences in the distribution of these molecules, consistent with the RGB expression results (Figure 8 and Figure 9).

## 4. Discussion

### 4.1. Analysis of Molecular Distribution

We have demonstrated, here, a method for quantifying the distribution of DNA, RNA, histone, and proteins other than histone in mammalian cells at different spatial scales using soft X-ray microscopy. Our results showed that DNA and histone were located in the nucleus, whereas RNA was clearly discriminated and found mainly in the cytoplasm. The images of DNA and RNA in the absorption images of the energy stack file (spect1) of the sample were extracted by the SVD method on aXis2000 using the spectra of DNA and RNA, each of which was measured at the photon energy regions of carbon, nitrogen, and oxygen K absorption edges and combined. The fraction of DNA in DNA plus RNA was applied to the quantitative image of nucleic acids in spect1 for the extraction of a quantitative DNA image. The energy stack file of DNA was then constructed and subtracted from spect1. The RNA image was obtained from the resulting stack file, spect2D. In the same manner, histone and BSA (as a representative of proteins other than histone) images were obtained. The residual stack file (spect3) contains spectra of molecules other than nucleic acids and proteins. In these processes, clear separation of the 1s–π * transition between N=C bond in nucleic acid at 399.6 eV and amide bond in protein at 401.4–401.5 eV (Figure 1) at the nitrogen K absorption edge region has been successfully applied, except to the areas shown in the ratio images of each residual image (spect3) to original absorption image (spect1) at higher than 0.3 in Figure 4 for the images of the cells and the nuclei excluding the chromosome as noted in the text for Figure 4 in the Results section.

### 4.2. Mass Thickness Distribution of DNA, RNA, Histone, and Proteins Other Than Histone

Two-dimensional distributions of DNA, RNA, histone, and proteins other than histone were estimated by the present method in all samples (Figure 5, Figure 6 and Figure 7 and Supplementary Figures S1–S3), showing that DNA and histone were localized in the nucleus, RNA mainly in the cytoplasm, and that proteins other than histone were omnipresent. These findings were clearly demonstrated in our analyses of RGB expressions (Figure 8 and Figure 9). The fact that no RNA was detected in the nucleus may be attributable to the low density distribution of RNA [30,31] compared with DNA and proteins, such that the signal from RNA may have been submerged in the total absorption [24]. Another explanation could relate to insufficient flux to the nucleus. We found that the density of histone was higher than that of DNA except for the case of the chromosome. RNA was widely distributed at low density in a range of areas. It should be noted that the RNA observed in the isolated nucleus was mainly located outside the nucleus (Figure 8).

### 4.3. Chromosome Structure

Interestingly, we found that DNA and RNA were separated within the chromosome. Distribution of mass thickness (Figure 6) and RGB expressions (Figure 8b and Figure 9b) showed that DNA and histone were located in the core position and RNA and other proteins were outside these. The RGB expression analysis suggested that proteins other than histone are at the outside and/or inside the core structure of DNA and histone, because the color for the position of DNA and histone is white, i.e., a combined color of red, green, and blue. It is not clear in Figure 8b that RNA covers the core of DNA and histone, probably because of the low level of content. It may be possible that RNA also surrounds the core of DNA and histone. These results are consistent with the finding that chromosomes contain a significant amount of RNA [32]. Furthermore, the combined image of DNA, RNA, and total proteins (Supplementary Figure S1) is similar to the absorption image of the chromosome (Figure 2b), indicating that these molecules are the major components. The total amount of DNA in the figure corresponding to the DNA content in the chromosome was estimated to be 0.73 pg (Table 1). Since the chromosome number of the cell is about 20, with a mixture of large and small (Figure 2b inset), the total amount of DNA in the CHO cell at M phase is estimated to be about 14 pg. This amount is in accord with estimates for mouse or human (about 13 pg) approximated from the size of haploid genome [33]. The amount of RNA, histone, and proteins other than histone, relative to DNA in the figure, was 0.48, 0.28, and 4.04, respectively.

### 4.4. Molecular Distribution in the Apoptotic Nucleus

We observed highly different structures in the apoptotic nucleus (Figure 7, Figure 8e and Figure 9e) compared to the intact nucleus (Figure 8d and Figure 9d, Supplementary Figure S3). In the apoptotic nucleus, most of the area is transparent and can be sufficiently exposed by X-rays. RNA was clearly observable, and was located in a different region than DNA and/or histone. Proteins other than histone were distributed in a wide area of the apoptotic nucleus, including alongside the RNA. We also found that DNA and histone were not always co-localized in the apoptotic nucleus.

### 4.5. Local Analysis of Absorption Spectra

In measuring local spectra in the absorbed image (spect1), we found similar spectral profiles in the CHO cell (Figure 10) and the chromosome (Figure 11), despite large differences in their compositions (Table 1). In the HeLa S3 cell and nuclei (Figure 12), differences in the spectral features are obvious between the cytoplasm (position 1) and the nuclei (positions 2 and 3). The difference is remarkable in the spectra at the carbon and oxygen K absorption edge regions. The reason for this difference remains to be studied, although the difference in the composition of nucleic acids and proteins (Table 1) may be one possible cause. It should be noted that a considerably similar structure is observed at the two positions in the apoptotic nucleus (positions 4 and 5 in Figure 12), although the distribution of nucleic acids and proteins is clearly different (Figure 7, Figure 8e and Figure 9e, Table 1). Further study of the distribution of molecules other than nucleic acids and proteins, including lipids and polysaccharides, will explain these questions.

### 4.6. X-Ray Spectromicroscopy

In principle, X-ray microscopy is an imaging method complementary to optical microscopy and electron microscopy, with respect to the resolution defined by diffraction limit and transmittance. Therefore, it has been expected to be developed as a good candidate method for investigating biological samples. To date, the most successful biological application of X-ray microscopy seems to be the 3D observation of frozen hydrated samples. However, there is another approach: spectromicroscopy, which allows for direct observation of the quantitative distribution of biological molecules [23,24].

Our findings showing that DNA and histone were located in the nucleus, whereas RNA was clearly discriminated and found mainly in the cytoplasm, suggesting that the method reported here has enormous potential for examining a wide range of component molecules. We have laid out a simple process for analyzing the distribution of DNA and RNA for the observed absorption data (spect1) by the SVD method and subtraction of the energy stack-files of these molecules from spect1; then, analysis of distributions of proteins as many kinds of proteins as the absorption spectra observed for the subtracted energy stack file by the SVD method and subtraction of the energy stack-files for these molecules from the subtracted data; and so on. This process could be continued for all the remaining molecules, even minor components in the sample, so far as the spectra for them could be prepared. However, limitations should be considered for the fidelity of the results.

To observe thick samples, such as the nucleus, it is not easy to supply a sufficient flux of the probing X-rays, as was shown in Figure 4, even for the analysis of major components. For the analysis of minor components, even more flux is required, with the serious accompanying problem of radiation damage. Radiation damage is unavoidable in principle, depending on the expected resolution. Contrast of the image is produced by the absorption of X-rays, the absorption of which destroys the chemical bond of the imaging molecules. The level of damage to the sample by X-rays depends on the imaging photon flux. It should be noted that even by phase contrast, there is extensive radiation damage produced by the absorption of probing X-rays directly or indirectly. The change in the structure occurs after the radiation damage by the movement, e.g., by diffusion, of the broken ends or fragments. The size and the movement of these broken materials may modify the resolution. Observation at high resolution or for thick samples requires higher flux and, thus, results in even more destruction of the structure. Therefore, a key limitation for observations is a trade-off between the disorganization of biological structures and the deterioration of resolution. To circumvent this problem, fixing the structure the structure has been proposed [34]. In the present results, X-ray imaging at the resolution of 100 to 400 nm may not change the structure in a dry state to a first-order approximation, although it is not clear to what extent radiation damage affected the structure of interest. The question remains to be studied.

For imaging minor molecules, there is the same problem as discussed above. Higher flux is required for the identification of minor fraction. The possibility of success for imaging is a matter of trade-off. Therefore, there is a limitation to observing and analyzing minor fractions of molecules in the sample. Observation may be successful in the case where thin-sliced samples are able to be prepared, although the preparation solely depends on the purpose of the observation.

Fixative may be a factor to modifying the X-ray image. In the present study, glutaraldehyde or a mixture of ethanol and acetic acid was used to fix the samples. These fixatives would not influence our results because they contain no nitrogen, and absorption peaks at the nitrogen K absorption edge region were applied for our analysis of quantitative distribution.

## 5. Conclusions

We have established a method for the analysis of biological data obtained with X-ray spectromicroscopy and estimated the quantitative distribution of DNA, RNA, histone, and proteins other than histone in a CHO cell, the cytoplasmic area of a HeLa cell, an apoptotic nucleus, and a chromosome for the first time. In particular, the results revealed the distinctive distribution of RNA in an apoptotic nucleus and a chromosome. The results demonstrated that we could determine the spatial distribution of molecules in cells by the quantitative treatment of X-ray absorption and suggested that the present method, in combination with X-ray spectromicroscopy, has an infinite potential for extending the analysis to the quantitative distribution of various molecules of interest in accordance with the technical improvement of an imaging system. In other words, the present method will shed light on novel cellular localization of biological molecules, which may lead to unique studies, e.g., elucidation of functional roles of microRNAs in chromosomal architecture or apoptotic structures.

## Figures and Tables

**Table 1:** Molecular contents of the regions of the positions shown in Figure 10, Figure 11 and Figure 12, and of the entire chromosome area.

Sample	Position	DNA	RNA	Histone	Other Proteins *	Total Content
		Content in the Area (fg)				(fg)
CHO	1	0.00	36.43	0.00	61.92	98.35
	2	0.54	50.40	2.12	186.24	239.30
	3	84.48	0.74	117.60	146.40	349.22
Chromosome	1	4.80	0.50	2.39	9.12	16.81
	2	1.14	1.01	0.15	9.36	11.66
	3	1.33	1.26	0.00	7.32	9.92
	4	0.20	0.12	0.22	3.15	3.68
	Whole Area	730	347	206	2952	4235
HeLa	1	0.01	13.15	0.41	48.48	62.06
	2	31.68	6.82	112.80	5.74	157.03
Intact Nucleus	3	18.00	0.04	33.55	6.48	58.07
Apoptotic Nucleus	4	3.63	10.51	9.37	21.17	44.68
	5	15.84	0.21	28.37	13.82	58.25

* Proteins other than histone.

## Supplementary Materials

Supplementary materials are available online at http://www.mdpi.com/2073-4409/8/2/164/s1.
