# Supplementary material for: Quantitative Distribution of DNA, RNA, Histone and Proteins Other than Histone in Mammalian Cells, Nuclei and a Chromosome at High Resolution Observed by Scanning Transmission Soft X-Ray Microscopy (STXM)
Source: Cells. 2019 Feb 16;8(2):164. doi: 10.3390/cells8020164 (PMC6406381; doi:10.3390/cells8020164)
Supplement: Supplementary file 1 [file cells-08-00164-s001.pdf]

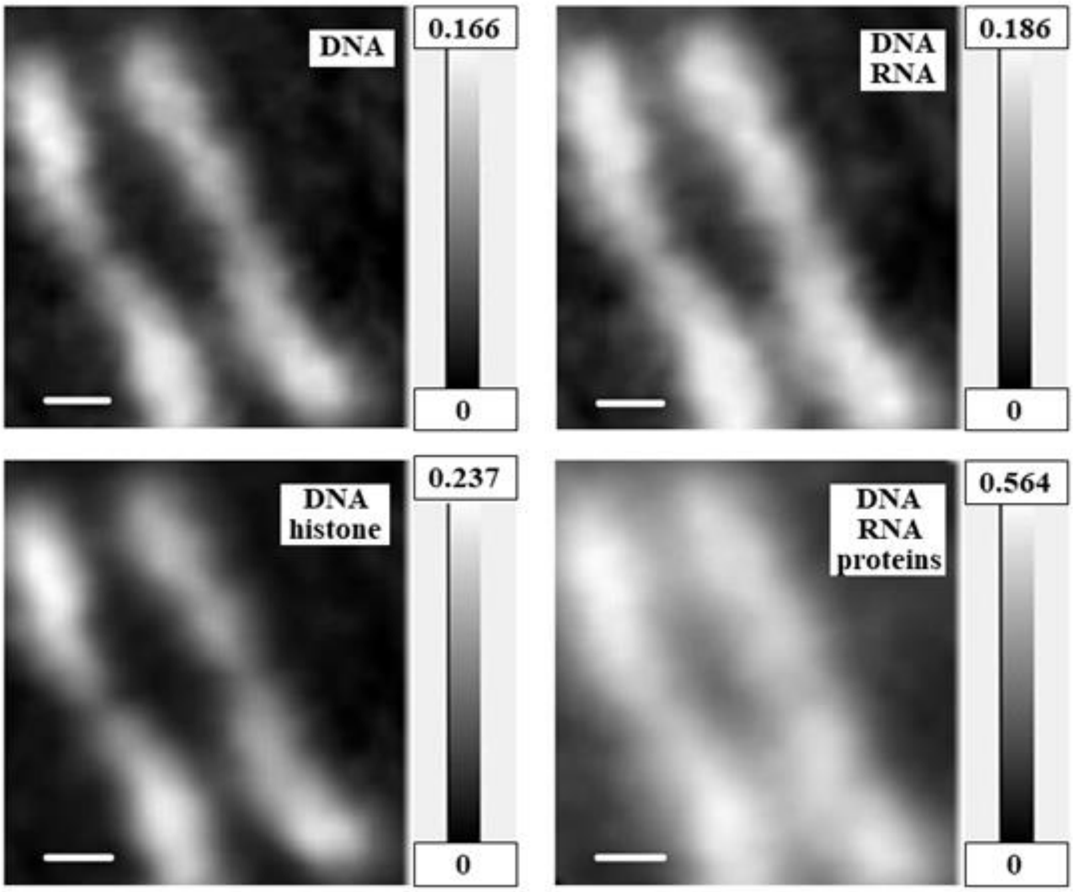

Figure S1. Mass thickness images for DNA, combined mass thickness images for DNA and RNA, DNA and histone, and DNA, RNA, and total proteins of the chromosome. Grayscales on right indicate units of pg/μm^2. Scale bars = 0.5 μm.

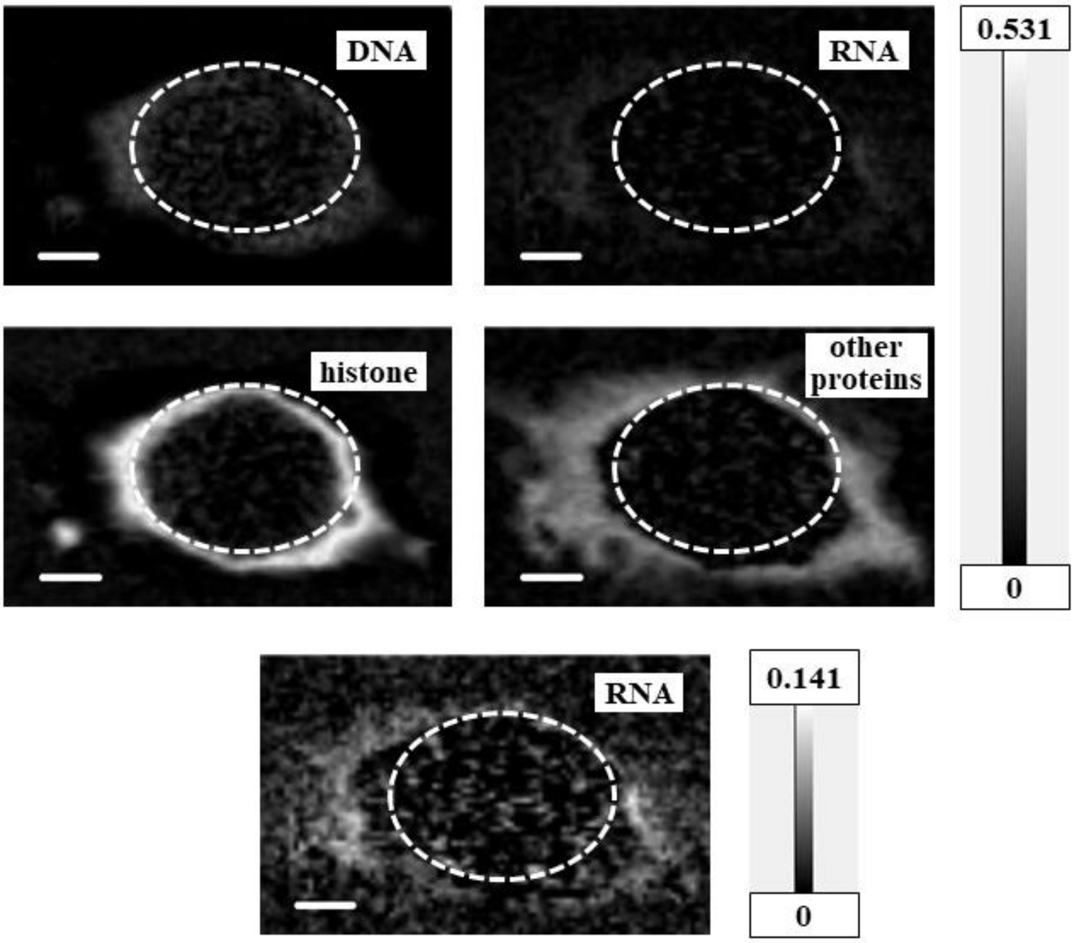

Figure S2. Mass thickness images for DNA, RNA, histone, and proteins other than histone of the HeLa S3 cell. Grayscales on right represent units of pg/μm^2—scale is consistent for top four images, and different for the bottom image to show the distribution of RNA more clearly. Scale bars represent 2 μm. Areas possibly unreliable for analysis are encircled by dashed lines.

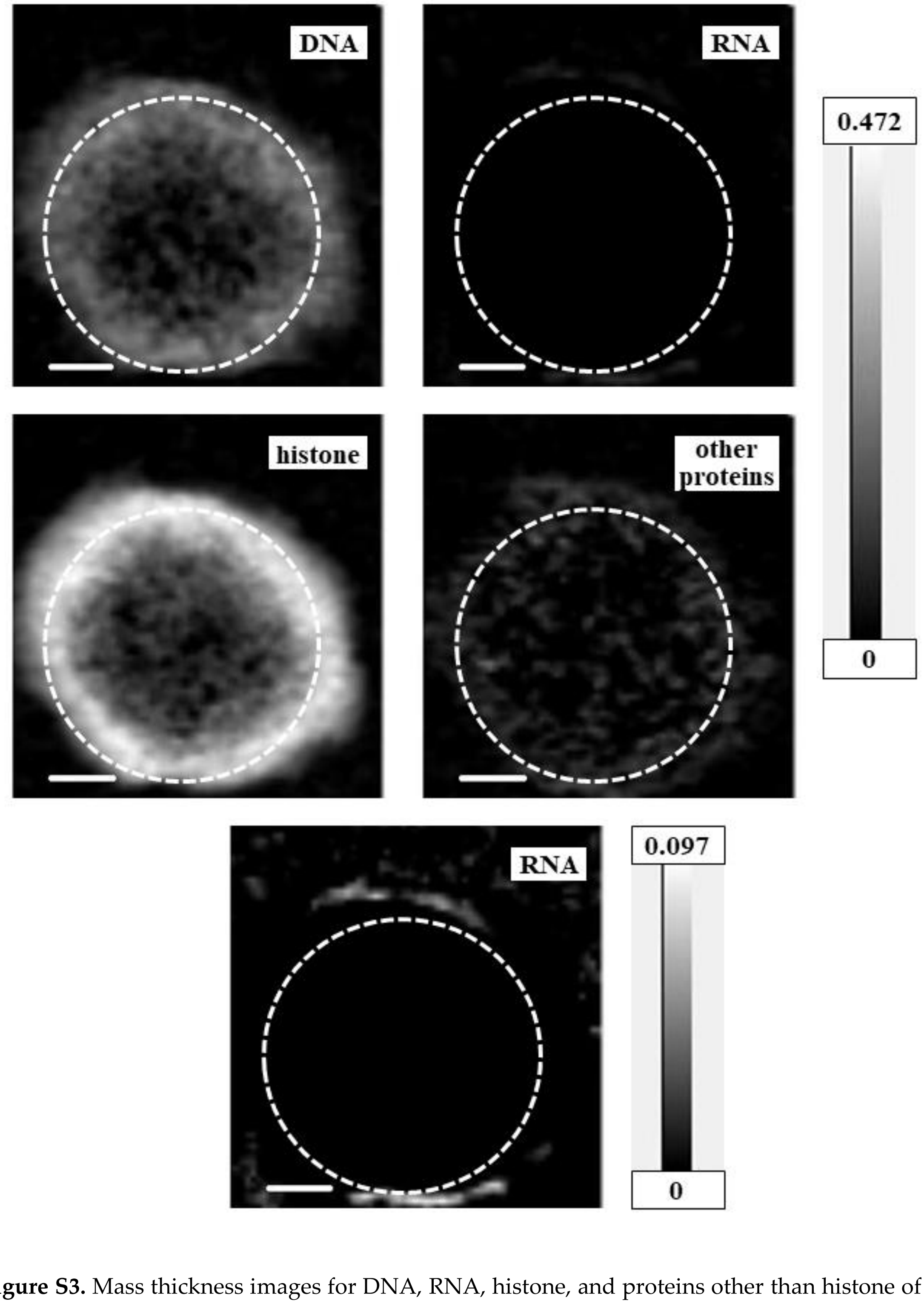

Figure S3. Mass thickness images for DNA, RNA, histone, and proteins other than histone of the isolated nucleus. Grayscales on right represent units of pg/μm^2—scale is consistent for top four images, and different for the bottom image to show the distribution of RNA more clearly. Scale bars represent 1 μm. Areas possibly unreliable for the analysis are encircled with dashed lines.
